# Early Childhood Exposure to Intimate Partner Violence and Developmental Vulnerability at Kindergarten: Linking Canadian Population-Level Administrative Data.

**DOI:** 10.23889/ijpds.v7i3.1861

**Published:** 2022-08-25

**Authors:** Janelle Boram Lee, Marni Brownell, Tracie Afifi, Lorna Turnbull, Marcelo Urquia, Nathan Nickel

**Affiliations:** 1 University of Calgary; 2 Manitoba Centre for Health Policy, University of Manitoba; 3 University of Manitoba

## Objectives

The objective was to examine the relationship between maternal intimate partner violence (IPV) victimization and children’s developmental health using linked population-wide administrative datasets. We examined developmental vulnerability (DV) at kindergarten of children exposed to maternal IPV victimization aged 0 to 5 using provincial prosecution records compared to unexposed counterparts.

## Approach

This retrospective cohort study linked administrative datasets (legal, health, education, social services) from the Population Research Data Repository at the Manitoba Centre for Health Policy. Exposed mother-child pairs with 1+ prosecution records of maternal IPV victimization during early childhood (child aged 0 to 5) between 2003-2018 in Manitoba (n = 5,728) were matched to unexposed pairs (1:3) based on sex/birthdate of child and neighbourhood income. DV at kindergarten was measured across 5 domains (physical, social, emotional, language/cognitive [LC], communication/general knowledge) using the Early Developmental Instrument (EDI). Children without eligible EDI scores were excluded. Multiple logistic regression models were conducted.

## Results

The cohort included 5321 children (exposed n=1365, unexposed n=3956). 32.98% of the cohort was developmentally vulnerable in one or more domains (1/+) and 19.60% was developmentally vulnerable in two or more domains (2/+). Unadjusted relationships between maternal IPV victimization from age 0 to 5 and developmental vulnerability at kindergarten were statistically significant across all 5 domains (e.g., LC OR=2.76 [2.36, 3.23]) and in 1/+ (OR=2.72 [2.39, 3.09]) as well as 2/+ (OR=2.89 [2.51, 3.34]) domains. After adjusting for covariates, children who were exposed to maternal IPV victimization from ages 0 to 5 had increased odds of being developmentally vulnerable in social competence (aOR=1.33 [1.07, 1.66]) and emotional maturity (aOR=1.29 [1.03, 1.62]), also in 2/+ domains (aOR=1.42 [1.15, 1.73]) at kindergarten, compared to unexposed counterparts.

## Conclusion and Relevance

The study provided Canadian population-wide evidence of the association between maternal IPV victimization and early childhood development, specifically later socio-emotional vulnerability. Interventions and support systems for this population of families should be developed and implemented, with an emphasis on mitigating long-term socio-emotional developmental risks in children exposed to IPV.

